# Trends in Socioeconomic Inequalities in Body Mass Index, Underweight and Obesity among English Children, 2007–2008 to 2011–2012

**DOI:** 10.1371/journal.pone.0147614

**Published:** 2016-01-26

**Authors:** James White, David Rehkopf, Laust Hvas Mortensen

**Affiliations:** 1 South East Wales Trials Unit (SEWTU), Cardiff University, Cardiff, United Kingdom; 2 Centre for the Development and Evaluation of Complex Interventions for Public Health Improvement (DECIPHer), Cardiff University, Cardiff, United Kingdom; 3 School of Medicine, Stanford University, Stanford, California, United States of America; 4 Faculty of Health and Medical Sciences, University of Copenhagen, Copenhagen, Denmark; Institute of Preventive Medicine, DENMARK

## Abstract

**Background:**

Socioeconomic inequalities in childhood obesity have been reported in most developed countries, with obesity more common in deprived groups. Whether inequalities are found in the prevalence of underweight, the rest of the body mass index (BMI) distribution, or have changed across time is not clear.

**Methods and Findings:**

The sample comprised 5,027,128 children on entry (4 to 5 years old) and leaving (10 to 11 years) state primary (elementary) school who participated in the National Child Measurement Programme (England, United Kingdom). We used area-level deprivation (Indices of Multiple Deprivation at the lower super output area) as a measure of socioeconomic deprivation. From 2007–2008 to 2011–2012 inequalities in obesity between the most compared to least deprived group increased (from 7.21% to 8.30%; *p*<0.001), whereas inequalities in the prevalence of underweight (1.50% to 1.21%; *p* = 0.15) were stable during this period. There were no differences by age group or by sex, but a three-way interaction suggested inequalities in obesity had increased at a faster rate for 10 to 11 year old girls, than 4 to 5 year old boys, (2.03% vs 0.07%; *p*<0.001 for interaction). Investigating inequalities across the distribution of zBMI showed increases in mean zBMI (0.18 to 0.23, *p*<0.001) could be attributed to increases in inequalities between the 50^th^ and 75^th^ centiles of BMI. Using the 2011 to 2012 population attributable risk estimates, if inequalities were halved, 14.04% (95% CI 14.00% to 14.07%) of childhood obesity could be avoided.

**Conclusions:**

Socioeconomic inequalities in childhood obesity and zBMI increased in England between 2007–2008 and 2011–2012. Inequalities in the prevalence of underweight did not change. Traditional methods of examining inequalities only at the clinical thresholds of overweight and obesity may have led the magnitude of inequalities in childhood BMI to be underestimated.

## Introduction

A socioeconomic gradient in childhood obesity has been reported in most middle to high income countries with a greater prevalence in more disadvantaged groups [1–4]. Although a levelling off of the obesity epidemic has been reported [5–7], the evidence on whether this has occurred at all socioeconomic levels is mixed, with studies reporting an increase [7–10], decrease [11] or, no change in inequalities across time [12–14]. Whether socioeconomic inequalities in the prevalence of childhood obesity are changing is important because both socioeconomic position [15] and obesity [16–17] track into adult life, and are independently associated with the development of cardiovascular disease risk factors [18–22]. Elevated cardiovascular risk factors, such as dyslipidaemia [23] and hypertension [24], are the predominant known cause of cardiovascular disease mortality. Despite substantial reductions in cardiovascular disease mortality since the 1960s, greater improvements have been made by the less socioeconomically deprived groups, such that these reductions were accompanied by a widening of inequalities [25–27].

The traditional modelling of inequalities in overweight and obesity, typically using logistic regression [10, 28], or the mean of BMI in linear models [10], has the result that inequalities occurring at other parts of the frequency distribution are ignored. While interpretation of this standard approach is simpler, it has two distinct limitations which sacrifices insights that can be made from examining inequalities across the entire BMI distribution. First, this approach neglects inequalities occurring in the prevalence of underweight. Underweight is important in preschool children as it is a risk factor for delayed cognitive development [29] and low academic attainment at school [30]. Secondly, a lack of change in inequalities in mean BMI may hide substantial differences in the proportion of obese- and underweight individuals which could offset each other. Despite these limitations of the standard approach, the evidence to date on inequalities in childhood body mass index has concentrated almost exclusively on only the higher centile thresholds of overweight and obesity [5–14]. The result is that there are gaps in our understanding on inequalities in underweight and across the BMI distribution. This information is needed to determine if policies to reduce health inequalities are working, or should change.

To address these gap in the literature, we undertook a time-trend analysis (2007–2008 to 2011–2012) on socioeconomic inequalities across the entire zBMI distribution, as well as estimating inequalities at the clinically important thresholds for underweight and obesity, in a nationally representative sample of children on entry (4 to 5 years old) and leaving (10 to 11 years) state primary (elementary) school.

## Methods

### Study Sample and Procedures

The National Child Measurement Programme (NCMP) is a school-based weight surveillance initiative of all children on entry to state primary (elementary) schooling (4 to 5 years old) and in year 6 (10 to 11 years) who reside in England. An opt-out system of school and parental consent is used in the NCMP. In 2011–2012, 93% of eligible children participated [31], compared with 88% in 2007–2008 [32], 90% in 2008–2009 [33], 91% in 2009–2010 [34], and 93% in 2010–2011 [35]. A detailed description of the NCMP is available elsewhere [32].

During a physical examination, height and weight were measured by trained personnel [36]. Children were weighed in light clothing without shoes to the nearest 0.1 kg, height measured to the nearest 0.1 cm using a free-standing stadiometer [36], and Body Mass Index was calculated (kg/m^2^). BMI z-scores were derived using the UK 1990 sex-specific BMI-for-age reference curves for children [37]. Underweight was defined ≤ 2^nd^ centile (used by the NCMP for population monitoring and clinical assessment), obesity ≥ 95^th^ centile (the population monitoring threshold for obesity prevalence used by the NCMP); as no agreed thresholds for morbid obesity exist we used the International Obesity Task Force (IOTF) cut-points (≥ 99.8^th^ centile) [38]. Parent-reported data were obtained from schools on children’s age and sex. Home postcode was used to derive 2007 Indices of Multiple Deprivation (IMD) scores (2010 IMD used from 2010–2011 onwards) at the Lower Super Output Area (LSOA) level, as a measure of socioeconomic deprivation in the child’s neighbourhood [39]. IMD scores were transformed into deciles.

### Statistical Analysis

Trends in inequalities in zBMI, underweight and obesity were examined from 2007–2008 through 2011–2012 using five time periods: 2007–2008, 2008–2009, 2009–2010, 2010–2011, and 2011–2012. Absolute inequalities we defined as the difference between children resident in IMD deciles one and ten. Trends in absolute inequalities in zBMI were tested using linear regression models with orthogonal polynomial contrasts. Trends in inequalities in the prevalence of underweight and obesity were examined using logistic models with orthogonal contrast matrices. Population attributable risks (PARs) were calculated by comparing logits in deciles one and ten to estimate the effect on prevalence if inequalities were eradicated, and the effect for deciles one to 5 to decile 10 for inequalities being halved.

Relative inequalities were tested using the relative index of inequality (RII). The RII is a summary measure which uses the whole socioeconomic distribution and is recommended for examining trends in inequalities across time [40]. We used log-binomial regression with a logarithmic link function to calculate RIIs [41,42]. Next, quantile regression was used to compare children in IMD decile 10 (most deprived) to with IMD decile 1 (least deprived) at half centile intervals across the zBMI distribution. We estimated standard errors using 100 bootstrap samples. We modelled the interaction between study year and IMD decile one versus ten to examine changes in inequalities across time. The estimates from quantile regression can be interpreted the same was as those from linear regression, with parameters representing the difference in zBMI between children in deciles 1 and 10 modelled at that percentile.

In preliminary analysis, we found a significant three-way interaction between the absolute inequalities in zBMI, study year and sex (*p*<0.001), and study year and school year (*p*<0.001). These interactions were also present for underweight (3-way inequalities, study year, sex interaction; *p*<0.001) and obesity (3-way inequalities, study year, sex interaction; *p*<0.001) so we conducted a combined then sex- and school year-stratified regression models. Continuous variables were examined with t-tests and categorical variables with the χ^2^ test. Analyses were conducted with Stata v13.0 and the package 'quantreg' v5.11 under R 3.0.2.

## Results

There were 5,105,126 children with measured height and weight in the National Child Measurement Programme 2007–2008 to 2011–2012. Of these, 1.48% (n = 75,706; 46,459 from 2007–2008) had a missing postcode such that a child residence could not be attributed to an LSOA and assigned an IMD score. We also removed 0.05% (n = 2,292) children who were six years of age at assessment as it was unclear why these children were assessed, or entered school a year late. The final analytical sample size was 5,027,128 (2,449,714 girls). Children included in the analysis had a slightly larger BMI z-score (0.43 vs. 0.42, *p* = 0.04), were more likely to be obese (14.35% vs. 14.04%, *p* = 0.01) but not underweight (1.17% vs. 1.16%, *p* = 0.64); however, absolute differences between the included and excluded were small with significance largely achieved owing to large sample size.

### Inequalities in zBMI and the Prevalence of Underweight and Obesity

In the overall population, in 2011 to 2012, the mean zBMI was 0.43 (95% CI, 0.43 to 0.43) and the prevalence of underweight and obesity were 1.21% (95% confidence interval [CI], 1.19 to 1.22%) and 14.00% (13.94 to 14.07%) respectively (S1 Table). zBMI was 0.23 (0.22 to 0.24) higher in children resident in the most compared to least deprived areas. The difference in the prevalence of underweight was 0.05% (0.04 to 0.06%) and obesity 8.30% (8.03 to 8.57%) lower among children resident in the least compared to the most deprived areas.

### Trends in Inequalities in zBMI and the Prevalence of Underweight and Obesity

In the overall population, unadjusted tests of trend indicated that from 2007–2008 to 2011–2012 inequalities in zBMI had widened by 0.04 (0.03 to 0.05, *p* for trend <0.001), obesity prevalence by 1.10% (0.70% to 1.48%, *p* for trend <0.001), and underweight stayed the same (0.00%, -0.15% to 0.13%, *p* for trend = 0.13). There was a dose response relationship between IMD decile and absolute inequalities in obesity (S2 Table). The trend in inequalities in underweight and obesity was replicated using the RII (S3 & S4 Tables). Population attributable risks indicated that according to the 2011 to 2012 estimates, if inequalities were eradicated, 36.28% (32.19% to 34.96%) equating to 53,488 cases, and if halved 14.04% (14.00% to 14.07%), or 20,700 cases of childhood obesity could be avoided.

### Trends in Inequalities according to Child’s Sex and Year Group

**Fig 1:**
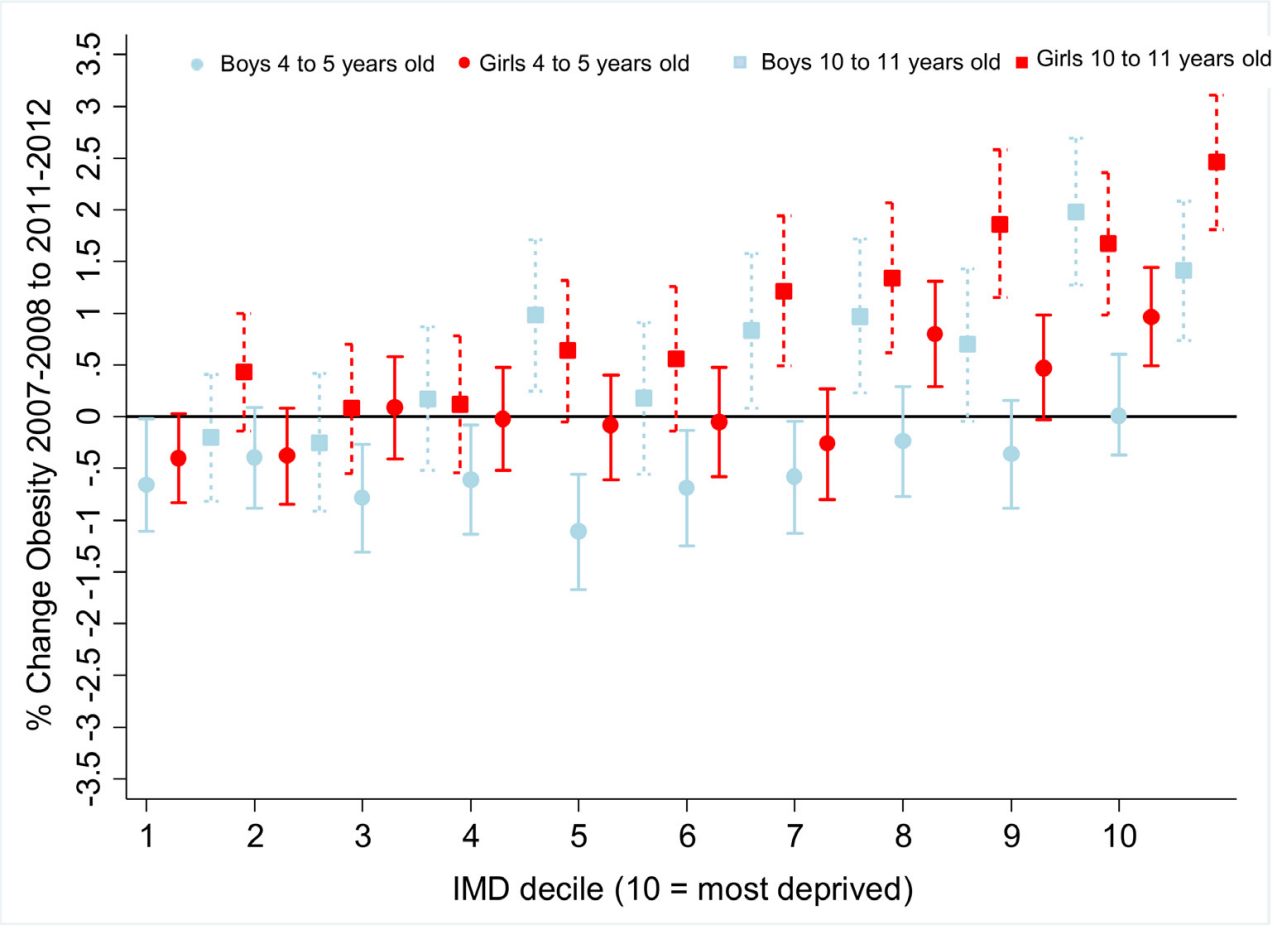
Trends for Obesity^a^ by Sex and Age Group by Area-level Deprivation^b^ England, 2007–2008 to 2011–2012^c^. ^a^ Obesity for youth aged 4 to 11 defined as having a body mass index (BMI) at or above the age and sex-specific 95^th^ centile on the UK 1990 Growth Reference. ^b^ Index of Multiple Deprivation (IMD) 2010 score derived from lower super output (LSOA) area of the child’s residence in 2007.^c^ Dashed lines indicate the 10 to 11 year old age group. ^d^ Data from the National Child Measurement Programme.

Sex- and school year-stratified stratified trend analyses are shown in Fig 1, S5–S7 Tables. Inequalities in zBMI (*p* = 0.66) and obesity (*p* = 0.51) had widened at a comparable rate for boys and girls. There was no change in inequalities in the prevalence of underweight, or any interaction by sex (*p* = 0.14). Neither was there a difference in the extent to which inequalities had changed across school years for zBMI (*p* = 0.32), or underweight (*p* = 0.13) and obesity (*p* = 0.81). Stratified by IMD decile and school year the prevalence of obesity was greater in the most compared to least deprived 10 to 11 year old girls than 4 to 5 year old boys (*p*<0.001 for interaction). Inequalities in the prevalence of underweight did not change when stratified IMD decile. These patterns of results were replicated when estimating inequalities using the RII (see S3 and S4 Tables).

### Inequalities Across the zBMI Distribution

**Fig 2:**
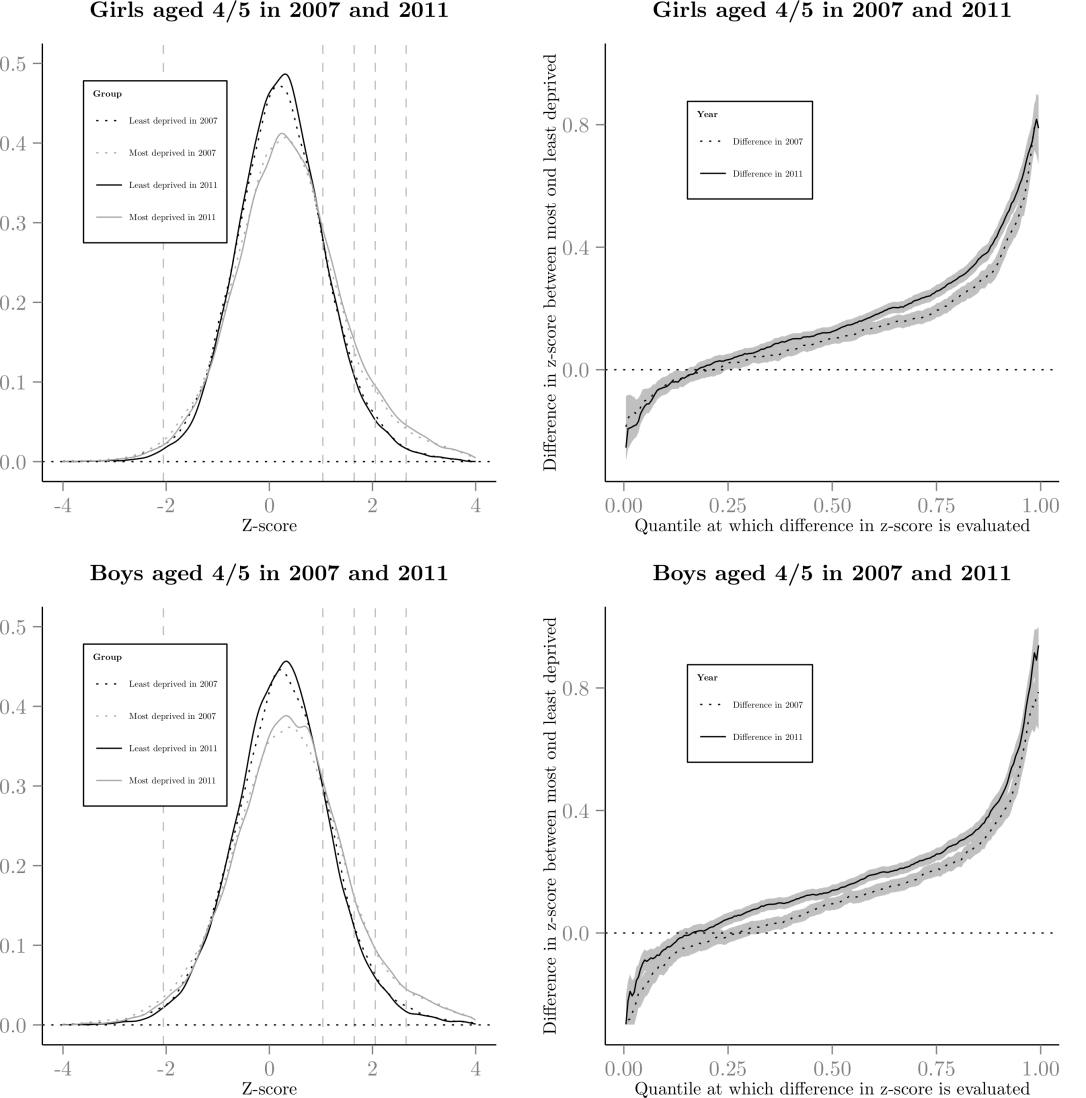
Kernel density graph of zBMI and the difference in zBMI (95% confidence interval for difference) for the least and most deprived groups in 2007 to 2008 and 2011 to 2012 for boys and girls 4 to 5 years of age. Vertical dashed lines on kernel density graphs show the UK 1990 definitions for underweight (2^nd^ centile), overweight (85^th^), obesity for population monitoring (95^th^), obesity for clinical classifications (98^th^) and morbid obesity (99.6^th^).

**Fig 3:**
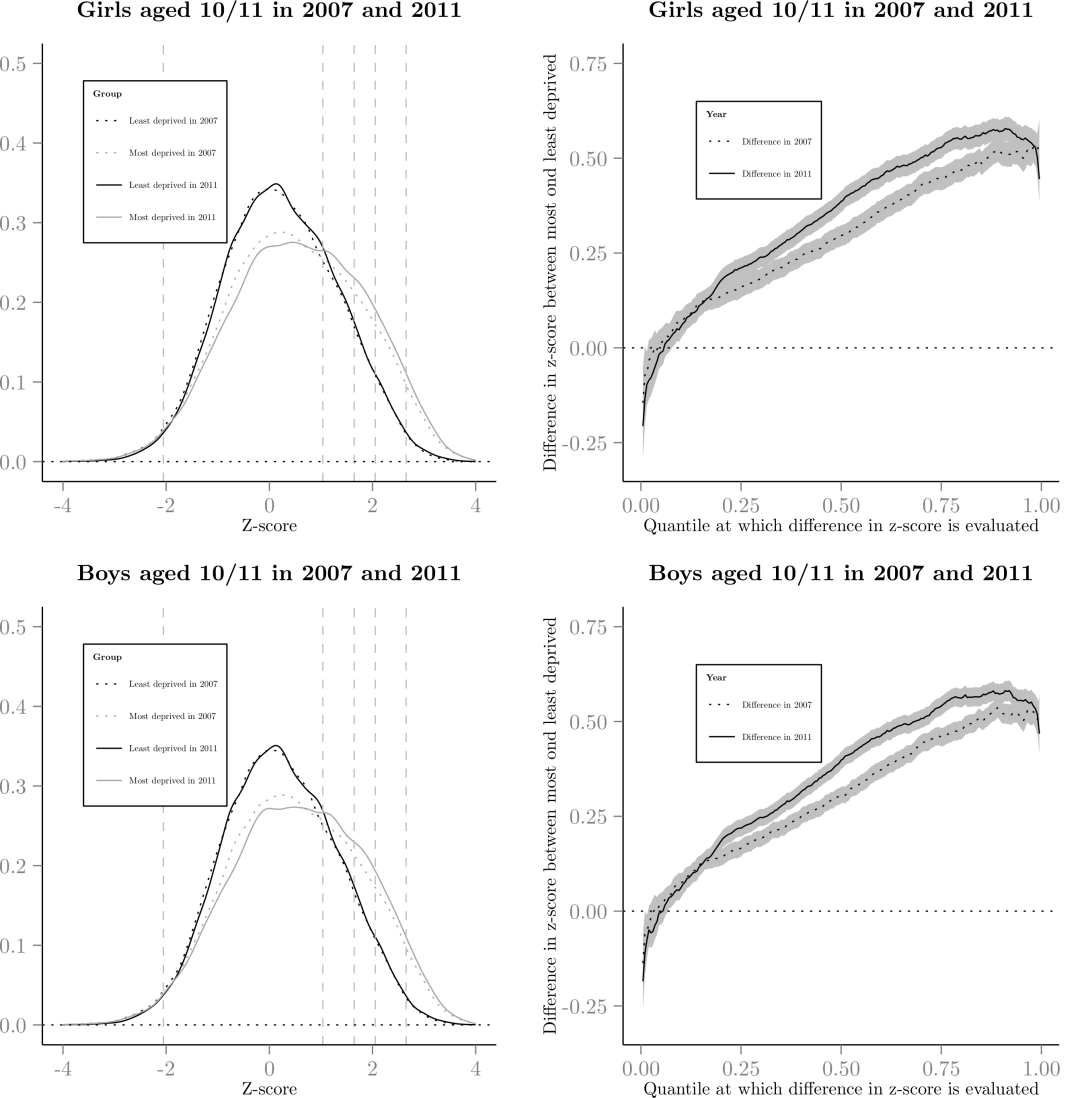
Kernel density graph of zBMI and the difference in zBMI (95% confidence interval for difference) for the least and most deprived groups in 2007 to 2008 and 2011 to 2012 for boys and girls 10 to 11 years of age. Vertical dashed lines on kernel density graphs show the UK 1990 definitions for underweight (2^nd^ centile), overweight (85^th^), obesity for population monitoring (95^th^), obesity for clinical classifications (98^th^) and morbid obesity (99.6^th^).

When taking a traditional analytic approach the linear regression estimates show a small increase in mean zBMI from 0.18 to 0.23 (*p*<0.001, S5 Table). The quantile regression examining inequalities at half centile intervals of the zBMI showed that below the 50^th^ centile the most deprived group had a lower zBMI and above the 50^th^ centile they had a higher zBMI, with larger inequalities found above the 50^th^ centile (see S5 and S6 Tables). The zBMI distribution was characterised by a shift in location and change in distribution shape (Figs 2 and 3). There was a lack of overlap between the 2007 and 2011 distributions indicating an increase in the number of children in the overweight to obese range. There was a little change in inequalities at the underweight range (1^st^ centile: *p* = 0.99; 2^nd^ centile: *p* = 0.61; 5^th^ centile: *p* = 0.77), but significant increases in inequalities at the 50^th^, 85^th^ (overweight centile threshold), and the obese range (90^th^, 91^st^, 95^th^, *p*<0.001), 98^th^ (*p* = 0.03), 99^th^ (*p* = 0.01) and 99.6^th^ centiles (*p*<0.001). Inequalities also widened at the 99.6^th^ centile at a faster rate for boys than girls (see Figs 2 and 3, and S5–S7 Tables). There was no difference in the trend in inequalities in zBMI at any quantile when stratified by school year, or school year and sex.

## Discussion

From 2007–2008 to 2011–2012, inequalities in the zBMI of children resident in the most compared to the least deprived areas in England increased. In 2011–2012, zBMI was 0.23, obesity 8.30% and underweight 0.05% higher in children resident in the most compared to the least deprived areas. The inequalities in mean zBMI widened by 0.04, obesity prevalence by 1.10% and there was no change in the prevalence of underweight. The changes in mean zBMI were associated with increases in inequality occurring in the normal to obese range (50^th^ to 98^th^ centiles).

The main strength of this paper is the analysis of 5,027,128 zBMI observations. This large sample size meant we had sufficient power to conduct a more comprehensive assessment of inequalities than has previously been published. We were able to examine sex and year group differences at half centile intervals. Inequalities were based on the IMD, a measure of socioeconomic deprivation in small areas, such that measures of household or parental socioeconomic disadvantage with a finer assessment of exposure to deprivation may have derived larger differences. Heights and weights were assessed objectively and not self-reported. This is important as BMI estimated from self-reports becomes progressively less accurate as body mass increases [43]. In sensitivity analysis, we found comparable trends using relative and absolute measures of inequality, strengthening confidence in our findings. In terms of weaknesses, it is worth noting that any test of trend depends on when is chosen as the start and end points of the examination. In this analysis, we selected 2007–2008 as the starting point because in 2006–2007 the NCMP had a lower participation rate 88%, which underestimated the prevalence of overweight and obesity in year 6 children [44]. The 2011–2012 data is the latest publically available dataset which has information on the IMD of the child’s residence, which restricted the trend analyses. The PAR estimates assume a direct causal relationship between socioeconomic inequalities and obesity. As there are few potential confounding factors assessed in the NCMP, the strength of the association and therefore number of cases avoided suggested by the PARs may be overestimated. As this analysis was restricted to one high income country, we cannot be sure that our results are representative of trends in socioeconomic inequalities in zBMI, obesity and underweight in other countries.

To the best of our knowledge, this is the first study to examine trends in inequalities across the entire distribution of zBMI. Our results are in agreement with those from other cohorts which found inequalities in the prevalence of obesity increased before 2006–2007 [8, 10], and during the period of study we examined (2007–2008 to 2011–2012) [45,46]. They replicate the findings from a diverse range of cohorts around the world which have shown a positive skewing of the BMI distribution in children and adolescents [47, 48]. We found substantial increases in inequalities were between the 50^th^ to 95^th^ centiles suggesting increases in inequalities were located where the distribution changed shape. This suggests more research is needed into interventions which can prevent children in this 50^th^ to 95^th^ centile range progressing into obesity are needed. Although a recently published systematic review suggested interventions to prevent and treat childhood obesity do not increase inequalities [49], an equitable effect at a population-level would maintain existing inequalities. Reducing inequalities may therefore require a proportionate universal approach [50]. This would mean that receipt of an intervention is proportionate to the degree of disadvantage, such that more children in deprived groups would receive programmes to prevent and/or treat obesity.

In conclusion, we found inequalities in zBMI and obesity increased, but underweight did not change over a short five year period in the UK [17]. Our analysis suggests that previous analyses have underestimated the magnitude of inequalities by not investigating those that occur under the threshold for overweight (85^th^ centile). As social inequalities established in early life track into early adulthood [17], achieving population-level reductions in the prevalence of obesity, as well as reductions in health inequalities will require more sophisticated implementation of policy to guard against further reduction in the health of children resident in the poorest areas. The routine monitoring of trends in underweight and obesity prevalence by socioeconomic position may help by increasing public and government awareness of the effectiveness of current UK government policies to reduce health inequalities.

## Supporting Information

S1 STROBE Checklist Trends in socioeconomic inequalities in Body Mass Index, Underweight and Obesity Among English Children, 2007–2008 to 2011–2012. (DOCX)

S1 Table Characteristics of participants in the National Child Measurement Programme, England, 2007–2012. (DOCX)

S2 Table Unadjusted Tests of Linear Trends for Obesity by Sex, Age and Area-level Deprivation, England, 2007–2012. (DOCX)

S3 Table Relative Index of Inequality for Inequalities in Underweight for Area-level Deprivation by Sex and Age, England, 2007–2012. (DOCX)

S4 Table Relative Index of Inequality for Inequalities in Obesity for Area-level Deprivation by Sex and Age, England, 2007–2012. (DOCX)

S5 Table Unadjusted Association between zBMI Obesity and Area-level Deprivation, England, 2007–2012. (DOCX)

S6 Table Unadjusted Association between zBMI Obesity and Area-level Deprivation in Boys, England, 2007–2012. (DOCX)

S7 Table Unadjusted Association between zBMI Obesity and Area-level Deprivation in Girls, England, 2007–2012. (DOCX)

## Abbreviations

NCMP: National Child Measurement Programme
IOTF: International Obesity Task Force
UKCRC: United Kingdom Clinical Research Collaboration
