# Supplementary material for: Trends in Socioeconomic Inequalities in Body Mass Index, Underweight and Obesity among English Children, 2007–2008 to 2011–2012
Source: PLoS One. 2016 Jan 26;11(1):e0147614. doi: 10.1371/journal.pone.0147614 (PMC4727904; doi:10.1371/journal.pone.0147614)
Supplement: S1 Table — (DOCX) [file pone.0147614.s002.docx]

**S1 Table. Characteristics of participants in the National Child Measurement Programme, England, 2007-2012**^a^

|  | | | **2007-2008** | **2008-2009** | | **2009-2010** | **2010-2011** | **2011-2012** | **P for trend** |
| --- | --- | --- | --- | --- | --- | --- | --- | --- | --- |
| Number of participants | | | 926,029 | | 996,061 | 1,017,364 | 1,035,095 | 1,052,579 |  |
| Sex | | |  | |  |  |  |  |  |
| Male | | | 51.38 (475,758) | | 51.33 (511,305) | 51.29 (521,761) | 51.21 (530,087) | 51.16 (538,503) |  |
| Female | | | 48.62 (450,271) | | 48.67 (484,756) | 48.71 (495,603) | 48.79 (505,008) | 48.83 (514,076) | 0.02 |
| School Year (Age) | | |  | |  |  |  |  |  |
| Reception | (4 to 5 years old) | | 49.14 (455,028) | | 50.46 (502,625) | 51.26 (521,503) | 52.18 (540,135) | 53.50 (563,162) |  |
| Year 6 | (10 to 11 years old) | | 50.86 (471,001) | | 49.54 (493,436) | 48.74 (495,861) | 47.82 (494,960) | 46.50 (489,417) | <0.001 |
| Area-level deprivation | | |  | |  |  |  |  |  |
| 1 (least deprived) | | | 10.04 (92,966) | | 9.95 (99,133) | 9.84 (100,076) | 9.67 (100,136) | 9.67 (101,744) |  |
| 2 | | | 9.30 (86,164) | | 9.34 (93,128) | 9.38 (95,378) | 9.29 (96,181) | 9.21 (96,936) |  |
| 3 | | | 8.81 (81,546) | | 8.97 (89,298) | 9.01 (91,714) | 8.89 (92,045) | 8.81 (92,827) |  |
| 4 | | | 8.60 (79,263) | | 8.80 (87,654) | 8.68 (88,351) | 8.72 (90,268) | 8.63 (90,870) |  |
| 5 | | | 8.76 (81,125) | | 8.98 (89,492) | 8.97 (91,231) | 8.89 (92,063) | 8.90 (93,702) |  |
| 6 | | | 9.03 (83,623) | | 9.25 (91,989) | 9.25 (94,065) | 9.24 (95,623) | 9.23 (97,191) |  |
| 7 | | | 9.49 (87,848) | | 9.61 (95,744) | 9.57 (97,395) | 9.67 (100,128) | 9.71 (102,213) |  |
| 8 | | | 10.39 (96,198) | | 10.41 (103,729) | 10.38 (105,629) | 10.50 (108,662) | 10.61 (111,682) |  |
| 9 | | | 11.69 (108,238) | | 11.57 (115,199) | 11.72 (119,223) | 11.79 (122,027) | 11.90 (125,242) |  |
| 10 (most deprived) | | | 13.93 (129,058) | | 13.12 (130,695) | 13.20 (134,302) | 13.33 (137,962) | 13.32 (140,172) | <0.001 |
| zBMI ^c^, Mean (SD) | | | 0.41 (1.14) | | 0.42 (1.12) | 0.44 (1.12) | 0.43 (1.12) | 0.43 (1.11) | <0.001 |
| Underweight, % (95% CI) ^c^ | | | 1.51 (1.49, 1.53) | | 1.29 (1.27, 1.31) | 1.24 (1.22, 1.26) | 1.28 (1.26, 1.30) | 1.21 (1.19, 1.22) | <0.001 |
| Obesity, % (95% CI) | | |  | |  |  |  |  |  |
|  | | 95^th^ centile ^c^ | 14.06 (13.99, 14.13) | | 13.91 (13.85, 13.98) | 14.17 (14.11, 14.24) | 14.03 (13.97, 14.10) | 14.00 (13.94, 14.07) | 0.88 |
|  | | 98^th^ centile ^c^ | 8.77 (8.71, 8.83) | | 8.63 (8.57, 8.68) | 8.82 (8.77, 8.88) | 8.76 (8.70, 8.81) | 8.78 (8.72, 8.83) | 0.05 |
| Morbid obesity ^d^, % (95% CI) | | | 1.44 (1.41, 1.46) | | 1.48 (1.46, 1.51) | 1.48 (1.46, 1.51) | 1.47 (1.45, 1.49) | 1.49 (1.47, 1.51) | <0.001 |

Data are % (n) of participants unless stated otherwise.

^a^ Data from the National Child Measurement Programme.

^b^ Index of Multiple Deprivation (IMD) 2010 score derived from lower super output (LSOA) area of the child’s residence.

^c^ Body Mass index z-scores (zBMI), underweight and obesity defined using the age and sex-specific UK 1990 Growth Reference.

^d^ Morbid obesity defined as having a BMI at or above the age and sex-specific cut-point defined by the International Obesity Task Force to approximate to the World Health Organisation adult definition of BMI ≥35.
