# Supplementary material for: Trends in Socioeconomic Inequalities in Body Mass Index, Underweight and Obesity among English Children, 2007–2008 to 2011–2012
Source: PLoS One. 2016 Jan 26;11(1):e0147614. doi: 10.1371/journal.pone.0147614 (PMC4727904; doi:10.1371/journal.pone.0147614)
Supplement: S2 Table — (DOCX) [file pone.0147614.s003.docx]

**S2 Table. Unadjusted Tests of Linear Trends for Obesity^a^ by Sex, Age and Area-level Deprivation^b^, England, 2007-2012^c^**

|  | | **% (95% CI)** | | | | | |  | **Change 2007-2008 to 2011-2012 (95% CI)** |
| --- | --- | --- | --- | --- | --- | --- | --- | --- | --- |
|  | | **2007-2008** | **2008-2009** | | **2009-2010** | **2010-2011** | **2011-2012** | ***P* for trend** |  |
| All | | 14. 06 (13. 99, 14. 13) | | 13. 91 (13. 85, 13. 98) | 14. 17 (14. 11, 14. 24) | 14. 03 (13. 97, 14. 10) | 14. 00 (13. 94, 14. 07) | 0. 88 | -0. 05 (-0. 15, 0. 05) |
| Sex | |  | |  |  |  |  |  |  |
| Male | | 15. 29 (15. 18, 15. 39) | | 15. 11 (15. 02, 15. 21) | 15. 31 (15. 21, 15. 40) | 15. 14 (15. 03, 15. 23) | 14. 94 (14. 84, 15. 03) | <0. 001 | -0. 27 (-0. 41, -0. 14) |
| Female | | 12. 76 (12. 66, 12. 85) | | 12. 65 (12. 56, 12. 74) | 12. 98 (12. 88, 13. 07) | 12. 88 (12. 78, 12. 97) | 13. 03 (12. 94, 13. 12) | <0. 001 | 0. 35 (0. 02, 0. 49) |
| School Year (Age) | |  | |  |  |  |  |  |  |
| Reception | 4 years | 9. 66 (9. 53, 9. 79) | | 9. 56 (9. 43, 9. 67) | 9. 72 (9. 60, 9. 83) | 9. 26 (9. 15, 9. 36) | 9. 30 (9. 19, 9. 40) | <0. 001 | -0. 04 (-0. 05, -0. 19) |
|  | 5 years | 9. 61 (9. 50, 9. 72) | | 9. 62 (9. 51, 9. 74) | 9. 99 (9. 82, 10. 04) | 9. 61 (9. 50, 9. 72) | 9. 70 (9. 60, 9. 82) | 0. 32 | 0. 10 (-0. 06, 0. 26) |
| Year 6 | 10 years | 18. 41 (18. 25, 18. 58) | | 18. 25 (18. 10, 18. 41) | 18. 82 (18. 67, 18. 97) | 19. 07 (18. 93, 19. 22) | 19. 20 (19. 05, 19. 34) | <0. 001 | 0. 78 (0. 56, 1. 00) |
|  | 11 years | 18. 28 (18. 13, 18. 42) | | 18. 36 (18. 21, 18. 51) | 18. 65 (18. 50, 18. 81) | 19. 03 (18. 87, 19. 20) | 19. 21 (19. 04, 19. 39) | <0. 001 | 0. 94 (0. 71, 1. 16) |
| Area-level deprivation  (IMD decile) | |  | |  |  |  |  |  |  |
| 1 (least deprived) | | 9. 80 (9. 61, 9. 99) | | 9. 63 (9. 45, 9. 81) | 9. 64 (9. 47, 9. 83) | 9. 48 (9. 31, 9. 67) | 9. 40 (9. 22, 9. 58) | 0. 002 | -0. 40 (-0. 70, -0. 13) |
| 2 | | 11. 09 (10. 88, 11. 30) | | 10. 56 (10. 36, 10. 76) | 11. 13 (10. 94, 11. 34) | 10. 90 (10. 70, 11. 10) | 10. 64 (10. 44, 10. 83) | 0. 85 | -0. 46 (-0. 74, -0. 17) |
| 3 | | 11. 84 (11. 62, 12. 06) | | 11. 58 (11. 37, 11. 79) | 11. 75 (11. 55, 11. 96) | 11. 69 (11. 48, 11. 90) | 11. 49 (11. 29, 11. 70) | 0. 93 | -0. 35 (-0. 65, -0. 05) |
| 4 | | 12. 37 (12. 14, 12. 60) | | 12. 43 (12. 21, 12. 65) | 12. 58 (12. 36, 12. 80) | 12. 27 (12. 05, 12. 49) | 12. 25 (12. 04, 12. 46) | 0. 24 | -0. 12 (-0. 43, 0. 19) |
| 5 | | 13. 58 (13. 34, 13. 82) | | 13. 40 (13. 18, 13. 62) | 13. 29 (13. 07, 13. 52) | 13. 08 (12. 87, 13. 30) | 13. 04 (12. 82, 13. 25) | 0. 001 | -0. 54 (-0. 86, -0. 22) |
| 6 | | 14. 40 (14. 16, 14. 63) | | 14. 22 (14. 00, 14. 57) | 14. 29 (14. 06, 14. 51) | 14. 27 (14. 05, 14. 50) | 14. 22 (14. 00, 14. 44) | 0. 42 | -0. 18 (-0. 50, 0. 15) |
| 7 | | 15. 29 (15. 05, 15. 52) | | 15. 20 (15. 78, 16. 22) | 15. 58 (15. 36, 15. 81) | 15. 21 (14. 99, 15. 43) | 15. 01 (14. 80, 15. 53) | 0. 12 | -0. 27 (-0. 60, 0. 05) |
| 8 | | 15. 98 (15. 75, 16. 21) | | 16. 00 (15. 75, 16. 21) | 16. 31 (16. 08, 16. 53) | 15. 99 (15. 78, 16. 22) | 16. 13 (15. 91, 16. 34) | 0. 43 | 0. 15 (-0. 16, 0. 47) |
| 9 | | 16. 87 (16. 65, 17. 09) | | 16. 89 (16. 68, 17. 10) | 17. 20 (16. 99, 17. 42) | 17. 19 (16. 98, 17. 41) | 17. 20 (17. 00, 17. 40) | 0. 006 | 0. 33 (0. 03, 0. 64) |
| 10 (most deprived) | | 17. 00 (16. 80, 17. 21) | | 17. 04 (16. 84, 17. 25) | 17. 55 (17. 35, 17. 75) | 17. 50 (17. 30, 17. 70) | 17. 77 (17. 51, 17. 91) | <0. 001 | 0. 70 (0. 41, 1. 00) |

^a^ Obesity for youth aged 4 to 11 defined as having a body mass index (BMI) at or above the age and sex-specific 95^th^ centile on the UK 1990 Growth Reference.

^b^ Index of Multiple Deprivation (IMD) 2010 score derived from lower super output (LSOA) area of the child’s residence.

^c^ Data from the National Child Measurement Programme.
