# Supplementary material for: Trends in Socioeconomic Inequalities in Body Mass Index, Underweight and Obesity among English Children, 2007–2008 to 2011–2012
Source: PLoS One. 2016 Jan 26;11(1):e0147614. doi: 10.1371/journal.pone.0147614 (PMC4727904; doi:10.1371/journal.pone.0147614)
Supplement: S3 Table — (DOCX) [file pone.0147614.s004.docx]

**S3 Table. Relative Index of Inequality for Inequalities in Underweight^a^ for Area-level Deprivation^b^ by Sex and Age, England, 2007-2012^c^**

|  | | **% (95% CI)** | | | | |  |
| --- | --- | --- | --- | --- | --- | --- | --- |
|  |  | **2007-2008** | **2008-2009** | **2009-2010** | **2010-2011** | **2011-2012** | ***P* for trend** |
| **All** |  | 1. 53 (1. 44, 1. 63) | 1. 58 (1. 48, 1. 68) | 1. 71 (1. 61, 1. 83) | 1. 78 (1. 67, 1. 89) | 1. 64 (1. 54, 1. 74) | 0. 006 |
| **Boys** |  | 1. 80 (1. 65, 1. 95) | 1. 69 (1. 55, 1. 85) | 1. 98 (1. 82, 2. 16) | 1. 96 (1. 80, 2. 13) | 1. 78 (1. 63, 1. 94) | 0. 33 |
| 4 to 5 years of age | | 1. 98 (1. 76, 2. 22) | 1. 50 (1. 33, 1. 69) | 1. 95 (1. 73, 2. 20) | 2. 04 (1. 81, 2. 29) | 1. 80 (1. 60, 2. 02) | 0. 56 |
| 10 to 11 years of age | | 1. 61 (1. 42, 1. 82) | 1. 95 (1. 72, 2. 22) | 2. 02 (1. 78, 2. 29) | 1. 88 (1. 66, 2. 13) | 1. 76 (1. 55, 2. 00) | 0. 43 |
| **Girls** |  | 1. 30 (1. 20, 1. 42) | 1. 46 (1. 34, 1. 69) | 1. 47 (1. 34, 1. 60) | 1. 61 (1. 47, 1. 75) | 1. 50 (1. 37, 1. 64) | 0. 005 |
| 4 to 5 years of age | | 1. 59 (1. 38, 1. 82) | 1. 64 (1. 41, 1. 90) | 2. 17 (1. 86, 2. 54) | 1. 93 (1. 67, 2. 24) | 1. 94 (1. 67, 2. 26) | 0. 01 |
| 10 to 11 years of age | | 1. 15 (1. 04, 1. 29) | 1. 37 (1. 23, 1. 53) | 1. 19 (1. 06, 1. 33) | 1. 45 (1. 30, 1. 61) | 1. 30 (1. 16, 1. 45) | 0. 10 |
|  |  |  |  |  |  |  |  |

^a^ Obesity for youth aged 4 to 11 defined as having a body mass index (BMI) at or below the age and sex-specific 2^nd^ centile on the UK 1990 Growth Reference.

^b^ Index of Multiple Deprivation (IMD) 2010 score derived from lower super output (LSOA) area of the child’s residence.

^c^ Data from the National Child Measurement Programme.
