# Supplementary material for: Trends in Socioeconomic Inequalities in Body Mass Index, Underweight and Obesity among English Children, 2007–2008 to 2011–2012
Source: PLoS One. 2016 Jan 26;11(1):e0147614. doi: 10.1371/journal.pone.0147614 (PMC4727904; doi:10.1371/journal.pone.0147614)
Supplement: S4 Table — (DOCX) [file pone.0147614.s005.docx]

**S4 Table. Relative Index of Inequality for Inequalities in Obesity^a^ for Area-level Deprivation^b^ by Sex and Age, England, 2007-2012^c^**

|  | | **% (95% CI)** | | | | |  |
| --- | --- | --- | --- | --- | --- | --- | --- |
|  |  | **2007-2008** | **2008-2009** | **2009-2010** | **2010-2011** | **2011-2012** | ***P* for trend** |
| **All** |  | 1. 89 (1. 85, 1. 92) | 1. 96 (1. 92, 1. 99) | 1. 99 (1. 96, 2. 02) | 2. 03 (1. 99, 2. 06) | 2. 08 (2. 04, 2. 11) | <0. 001 |
| **Boys** |  | 1. 80 (1. 75, 1. 84) | 1. 96 (1. 92, 1. 99) | 1. 99 (1. 96, 2. 02) | 2. 03 (1. 99, 2. 06) | 2. 08 (2. 04, 2. 11) | <0. 001 |
| 4 to 5 years of age | | 1. 84 (1. 76, 1. 92) | 1. 97 (1. 89, 2. 05) | 1. 98 (1. 90, 2. 06) | 2. 06 (1. 98, 2. 15) | 2. 01 (1. 94, 2. 09) | 0. 001 |
| 10 to 11 years of age | | 1. 77 (1. 72, 1. 82) | 1. 76 (1. 71, 1. 81) | 1. 83 (1. 78, 1. 88) | 1. 85 (1. 80, 1. 90) | 1. 90 (1. 85, 1. 96) | <0. 001 |
| **Girls** |  | 2. 00 (1. 95, 2. 06) | 2. 13 (2. 07, 2. 19) | 2. 12 (2. 07, 2. 18) | 2. 12 (2. 07, 2. 18) | 2. 24 (2. 18, 2. 29) | <0. 001 |
| 4 to 5 years of age | | 1. 95 (1. 86, 2. 05) | 2. 12 (2. 03, 2. 22) | 2. 10 (2. 01, 2. 19) | 2. 16 (2. 06, 2. 25) | 2. 22 (2. 13, 2. 32) | <0. 001 |
| 10 to 11 years of age | | 2. 02 (1. 96, 2. 09) | 2. 12 (2. 06, 2. 19) | 2. 13 (2. 06, 2. 20) | 2. 15 (2. 08, 2. 21) | 2. 24 (2. 16, 2. 31) | <0. 001 |
|  |  |  |  |  |  |  |  |

^a^ Obesity for youth aged 4 to 11 defined as having a body mass index (BMI) at or above the age and sex-specific 95^th^ centile on the UK 1990 Growth Reference.

^b^ Index of Multiple Deprivation (IMD) 2010 score derived from lower super output (LSOA) area of the child’s residence.

^c^ Data from the National Child Measurement Programme.
