# Supplementary material for: Trends in Socioeconomic Inequalities in Body Mass Index, Underweight and Obesity among English Children, 2007–2008 to 2011–2012
Source: PLoS One. 2016 Jan 26;11(1):e0147614. doi: 10.1371/journal.pone.0147614 (PMC4727904; doi:10.1371/journal.pone.0147614)
Supplement: S5 Table — (DOCX) [file pone.0147614.s006.docx]

**S5 Table. Unadjusted Association between zBMI Obesity^a^ and Area-level Deprivation^b^, England, 2007-2012^c^**

|  | **% (95% CI)** | | | | | |  |
| --- | --- | --- | --- | --- | --- | --- | --- |
|  | **2007-2008** | **2008-2009** | | **2009-2010** | **2010-2011** | **2011-2012** | ***P* for trend** |
| Mean ^d^ | 0. 18 (0. 18, 0. 19) | | 0. 19 (0. 18, 0. 20) | 0. 20 (0. 19, 0. 21) | 0. 22 (0. 21, 0. 23) | 0. 23 (0. 22, 0. 24) | <. 001 |
| zBMI Percentile ^e^ |  | |  |  |  |  |  |
| 1^st^ | -0. 17 (-0. 21, -0. 14) | | -0. 22 (-0. 26, -0. 19) | -0. 19 (-0. 22, -0. 16) | -0. 22 (-0. 25, -0. 18) | -0. 18 (-0. 22, -0. 14) | 0. 99 |
| 2^st^ | -0. 13 (-0. 16, -0. 10) | | -0. 14 (-0. 17, -0. 11) | -0. 14 (-0. 17, -0. 12) | -0. 14 (-0. 17, -0. 12) | -0. 14 (-0. 17, -0. 11) | 0. 61 |
| 5^th^ | -0. 08 (-0. 09, -0. 06) | | -0. 06 (-0. 07, -0. 04) | -0. 07 (-0. 09, -0. 05) | -0. 06 (-0. 08, -0. 04) | -0. 07 (-0. 09, -0. 05) | 0. 77 |
| 10^th^ | -0. 02 (-0. 03, -0. 01) | | -0. 01 (-0. 03, 0. 01) | -0. 02 (-0. 03, -0. 01) | -0. 01 (-0. 03, 0. 01) | -0. 01 (-0. 03, -0. 01) | 0. 57 |
| 50^th^ | 0. 16 (0. 15, 0. 17) | | 0. 17 (0. 16, 0. 17) | 0. 17 (0. 16, 0. 18) | 0. 19 (0. 18, 0. 20) | 0. 20 (0. 19, 0. 21) | <0. 001 |
| 85^th^ | 0. 40 (0. 39, 0. 42) | | 0. 41 (0. 39, 0. 42) | 0. 43 (0. 42, 0. 45) | 0. 46 (0. 44, 0. 48) | 0. 47 (0. 45, 0. 48) | <0. 001 |
| 90^th^ | 0. 47 (0. 45, 0. 49) | | 0. 47 (0. 45, 0. 49) | 0. 51 (0. 49, 0. 52) | 0. 51 (0. 50, 0. 53) | 0. 54 (0. 52, 0. 55) | <0. 001 |
| 91^st^ | 0. 47 (0. 46, 0. 49) | | 0. 47 (0. 46, 0. 50) | 0. 51 (0. 50, 0. 53) | 0. 52 (0. 51, 0. 54) | 0. 54 (0. 53, 0. 56) | <0. 001 |
| 95^th^ | 0. 51 (0. 49, 0. 53) | | 0. 52 (0. 50, 0. 55) | 0. 56 (0. 54, 0. 58) | 0. 57 (0. 55, 0. 59) | 0. 58 (0. 56, 0. 60) | <0. 001 |
| 98^th^ | 0. 55 (0. 52, 0. 58) | | 0. 55 (0. 53, 0. 57) | 0. 58 (0. 55, 0. 61) | 0. 56 (0. 54, 0. 59) | 0. 59 (0. 57, 0. 62) | 0. 03 |
| 99^th^ | 0. 56 (0. 53, 0. 60) | | 0. 54 (0. 51, 0. 58) | 0. 60 (0. 57, 0. 64) | 0. 60 (0. 57, 0. 64) | 0. 61 (0. 58, 0. 64) | 0. 01 |
| 99. 6^th^ | 0. 60 (0. 54, 0. 65) | | 0. 54 (0. 47, 0. 59) | 0. 66 (0. 61, 0. 73) | 0. 64 (0. 59, 0. 70) | 0. 68 (0. 62, 0. 73) | <0. 001 |

**^a^** zBMI calculated using the UK 1990 Growth Reference.

^b^ Index of Multiple Deprivation (IMD) 2010 decile one (least deprived) versus ten (most deprived) from the lower super output (LSOA) area of the child’s residence.

^c^ Data from the National Child Measurement Programme.

^d^ Estimated using ordinary least squares regression.

^e^ Estimated using quantile regression with decile one as the reference category; 95% confidence intervals calculated using bootstrapping with 100 replications.
