# Supplementary material for: Trends in Socioeconomic Inequalities in Body Mass Index, Underweight and Obesity among English Children, 2007–2008 to 2011–2012
Source: PLoS One. 2016 Jan 26;11(1):e0147614. doi: 10.1371/journal.pone.0147614 (PMC4727904; doi:10.1371/journal.pone.0147614)
Supplement: S6 Table — (DOCX) [file pone.0147614.s007.docx]

**S6 Table. Unadjusted Association between zBMI Obesity^a^ and Area-level Deprivation^b^ in Boys, England, 2007-2012^c^**

|  | **% (95% CI)** | | | | | |  |
| --- | --- | --- | --- | --- | --- | --- | --- |
|  | **2007-2008** | **2008-2009** | | **2009-2010** | **2010-2011** | **2011-2012** | ***P* for trend** |
| **4 to 5 years of age** |  | |  |  |  |  |  |
| Mean ^d^ | 0. 12 (0. 10, 0. 14) | | 0. 15 (0. 14, 0. 17) | 0. 15 (0. 13, 0. 16) | 0. 16 (0. 15, 0. 18) | 0. 18 (0. 16, 0. 19) | <0. 001 |
| zBMI Percentile ^e^ |  | |  |  |  |  |  |
| 1^st^ | -0. 28 (-0. 37, -0. 19) | | -0. 23 (-0. 32, -0. 15) | -0. 29 (-0. 36, -0. 22) | -0. 29 (-0. 38, -0. 20) | -0. 22 (-0. 28, -0. 17) | 0. 67 |
| 2^st^ | -0. 26 (-0. 33, -0. 19) | | -0. 17 (-0. 21, -0. 11) | -0. 21 (-0. 26, -0. 16) | -0. 21 (-0. 29, -0. 15) | -0. 21 (-0. 26, -0. 15) | 0. 31 |
| 5^th^ | -0. 17 (-0. 21, -0. 13) | | -0. 09 (-0. 13, -0. 05) | -0. 10 (-0. 14, -0. 07) | -0. 11 (-0. 15, -0. 07) | -0. 09 (-0. 12, -0. 05) | 0. 06 |
| 10^th^ | -0. 10 (-0. 13, -0. 07) | | -0. 03 (-0. 06, -0. 01) | -0. 06 (-0. 08, -0. 03) | -0. 07 (-0. 10, -0. 04) | -0. 05 (-0. 08, -0. 03) | 0. 34 |
| 50^th^ | 0. 10 (0. 07, 0. 12) | | 0. 13 (0. 11, 0. 14) | 0. 11 (0. 09, 0. 13) | 0. 14 (0. 12, 0. 15) | 0. 13 (0. 12, 0. 16) | 0. 002 |
| 85^th^ | 0. 29 (0. 26, 0. 31) | | 0. 32 (0. 29, 0. 34) | 0. 32 (0. 29, 0. 35) | 0. 34 (0. 31, 0. 38) | 0. 34 (0. 31, 0. 37) | <0. 001 |
| 90^th^ | 0. 38 (0. 34, 0. 41) | | 0. 38 (0. 35, 0. 41) | 0. 39 (0. 36, 0. 43) | 0. 43 (0. 40, 0. 47) | 0. 43 (0. 39, 0. 47) | 0. 001 |
| 91^st^ | 0. 39 (0. 35, 0. 43) | | 0. 41 (0. 37, 0. 44) | 0. 42 (0. 38, 0. 45) | 0. 45 (0. 41, 0. 49) | 0. 46 (0. 42, 0. 49) | 0. 001 |
| 95^th^ | 0. 54 (0. 49, 0. 58) | | 0. 54 (0. 48, 0. 59) | 0. 58 (0. 54, 0. 63) | 0. 60 (0. 55, 0. 64) | 0. 61 (0. 56, 0. 66) | 0. 002 |
| 98^th^ | 0. 74 (0. 66, 0. 82) | | 0. 72 (0. 65, 0. 79) | 0. 81 (0. 73, 0. 88) | 0. 82 (0. 73, 0. 90) | 0. 86 (0. 80, 0. 93) | 0. 001 |
| 99^th^ | 0. 78 (0. 66, 0. 89) | | 0. 81 (0. 72, 0. 90) | 0. 85 (0. 76, 0. 94) | 0. 88 (0. 77, 0. 99) | 0. 89 (0. 76, 1. 02) | 0. 12 |
| 99. 6^th^ | 0. 77 (0. 63, 0. 92) | | 0. 65 (0. 49, 0. 81) | 0. 85 (0. 75, 0. 95) | 0. 86 (0. 74, 0. 98) | 0. 97 (0. 86, 1. 08) | 0. 002 |
| **10 to 11 years of age** |  | |  |  |  |  |  |
| Mean ^d^ | 0. 21 (0. 19, 0. 23) | | 0. 21 (0. 19, 0. 23) | 0. 24 (0. 22, 0. 26) | 0. 26 (0. 24, 0. 28) | 0. 26 (0. 24, 0. 28) | <. 001 |
| 1^st^ | -0. 21 (-0. 29, -0. 13) | | -0. 25 (-0. 31, -0. 19) | -0. 28 (-0. 35, -0. 20) | -0. 28 (-0. 36, -0. 20) | -0. 21 (-0. 28, -0. 14) | 0. 63 |
| 2^st^ | -0. 13 (-0. 19, -0. 07) | | -0. 18 (-0. 25, -0. 12) | -0. 20 (-0. 25, -0. 15) | -0. 21 (-0. 27, -0. 15) | -0. 15 (-0. 21, -0. 10) | 0. 43 |
| 5^th^ | -0. 06 (-0. 10, 0. 02) | | -0. 10 (-0. 13, -0. 06) | -0. 10 (-0. 14, -0. 06) | -0. 10 (-0. 15, -0. 07) | -0. 09 (-0. 13, -0. 05) | 0. 37 |
| 10^th^ | -0. 03 (-0. 07, -0. 01) | | -0. 05 (-0. 08, -0. 03) | -0. 04 (-0. 08, -0. 02) | -0. 01 (-0. 05, 0. 02) | -0. 04 (-0. 07, -0. 01) | 0. 50 |
| 50^th^ | 0. 20 (0. 18, 0. 24) | | 0. 21 (0. 17, 0. 24) | 0. 23 (0. 21, 0. 26) | 0. 27 (0. 25, 0. 30) | 0. 28 (0. 25, 0. 31) | <0. 001 |
| 85^th^ | 0. 47 (0. 44, 0. 50) | | 0. 46 (0. 43, 0. 48) | 0. 53 (0. 50, 0. 56) | 0. 55 (0. 52, 0. 57) | 0. 56 (0. 53, 0. 58) | <0. 001 |
| 90^th^ | 0. 47 (0. 43, 0. 50) | | 0. 48 (0. 44, 0. 51) | 0. 52 (0. 49, 0. 55) | 0. 53 (0. 51, 0. 57) | 0. 55 (0. 51, 0. 58) | <0. 001 |
| 91^st^ | 0. 46 (0. 43, 0. 50) | | 0. 49 (0. 45, 0. 53) | 0. 53 (0. 49, 0. 56) | 0. 54 (0. 51, 0. 57) | 0. 54 (0. 50, 0. 57) | <0. 001 |
| 95^th^ | 0. 43 (0. 40, 0. 47) | | 0. 48 (0. 44, 0. 52) | 0. 50 (0. 47, 0. 53) | 0. 50 (0. 47, 0. 53) | 0. 50 (0. 47, 0. 54) | 0. 001 |
| 98^th^ | 0. 41 (0. 37, 0. 46) | | 0. 45 (0. 40, 0. 49) | 0. 46 (0. 42, 0. 50) | 0. 43 (0. 40, 0. 46) | 0. 44 (0. 40, 0. 48) | 0. 53 |
| 99^th^ | 0. 38 (0. 34, 0. 42) | | 0. 41 (0. 36, 0. 46) | 0. 44 (0. 39, 0. 48) | 0. 39 (0. 34, 0. 44) | 0. 42 (0. 37, 0. 47) | 0. 35 |
| 99. 6^th^ | 0. 28 (0. 21, 0. 35) | | 0. 33 (0. 26, 0. 41) | 0. 42 (0. 37, 0. 47) | 0. 33 (0. 28, 0. 38) | 0. 39 (0. 34, 0. 45) | 0. 02 |

**^a^** zBMI calculated using the UK 1990 Growth Reference.

^b^ Index of Multiple Deprivation (IMD) 2010 decile one (least deprived) versus ten (most deprived) from the lower super output (LSOA) area of the child’s residence.

^c^ Data from the National Child Measurement Programme.

^d^ Estimated using ordinary least squares regression.

^e^ Estimated using quantile regression with decile one as the reference category; 95% confidence intervals calculated using bootstrapping with 100 replications.
