# Supplementary material for: Trends in Socioeconomic Inequalities in Body Mass Index, Underweight and Obesity among English Children, 2007–2008 to 2011–2012
Source: PLoS One. 2016 Jan 26;11(1):e0147614. doi: 10.1371/journal.pone.0147614 (PMC4727904; doi:10.1371/journal.pone.0147614)
Supplement: S7 Table — (DOCX) [file pone.0147614.s008.docx]

**S7 Table. Unadjusted Association between zBMI Obesity^a^ and Area-level Deprivation^b^ in Girls, England, 2007-2012^c^**

|  | **% (95% CI)** | | | | | |  |
| --- | --- | --- | --- | --- | --- | --- | --- |
|  | **2007-2008** | **2008-2009** | | **2009-2010** | **2010-2011** | **2011-2012** | ***P* for trend** |
| **4 to 5 years of age** |  | |  |  |  |  |  |
| Mean ^d^ | 0. 13 (0. 11, 0. 15) | | 0. 13 (0. 11, 0. 15) | 0. 14 (0. 12, 0. 15) | 0. 15 (0. 13, 0. 17) | 0. 17 (0. 15, 0. 18) | 0. 02 |
| zBMI Percentile ^e^ |  | |  |  |  |  |  |
| 1^st^ | -0. 16 (-0. 24, -0. 08) | | -0. 23 (-0. 31, -0. 16) | -0. 21 (-0. 28, -0. 14) | -0. 17 (-0. 24, -0. 11) | -0. 19 (-0. 27, -0. 11) | 0. 58 |
| 2^st^ | -0. 14 (-0. 20, -0. 08) | | -0. 15 (-0. 20, -0. 10) | -0. 19 (-0. 25, -0. 14) | -0. 14 (-0. 20, -0. 09) | -0. 19 (-0. 24, -0. 14) | 0. 30 |
| 5^th^ | -0. 10 (-0. 14, -0. 06) | | -0. 07 (-0. 11, -0. 04) | -0. 10 (-0. 14, -0. 06) | -0. 08 (-0. 12, -0. 04) | -0. 12 (-0. 15, -0. 09) | 0. 30 |
| 10^th^ | -0. 05 (-0. 08, -0. 02) | | -0. 05 (-0. 08, -0. 03) | -0. 06 (-0. 08, -0. 03) | -0. 05 (-0. 07, -0. 02) | -0. 06 (-0. 08, -0. 03) | 0. 77 |
| 50^th^ | 0. 10 (0. 08, 0. 12) | | 0. 09 (0. 07, 0. 11) | 0. 11 (0. 09, 0. 13) | 0. 11 (0. 09, 0. 13) | 0. 12 (0. 11, 0. 14) | 0. 02 |
| 85^th^ | 0. 28 (0. 25, 0. 31) | | 0. 29 (0. 26, 0. 31) | 0. 31 (0. 28, 0. 34) | 0. 31 (0. 28, 0. 33) | 0. 35 (0. 34, 0. 38) | <0. 001 |
| 90^th^ | 0. 35 (0. 32, 0. 39) | | 0. 37 (0. 34, 0. 41) | 0. 38 (0. 36, 0. 42) | 0. 40 (0. 37, 0. 43) | 0. 45 (0. 43, 0. 47) | <0. 001 |
| 91^st^ | 0. 39 (0. 35, 0. 43) | | 0. 40 (0. 37, 0. 44) | 0. 42 (0. 38, 0. 45) | 0. 43 (0. 39, 0. 46) | 0. 48 (0. 45, 0. 51) | <0. 001 |
| 95^th^ | 0. 51 (0. 47, 0. 56) | | 0. 52 (0. 48, 0. 57) | 0. 56 (0. 52, 0. 60) | 0. 57 (0. 52, 0. 62) | 0. 61 (0. 56, 0. 65) | 0. 001 |
| 98^th^ | 0. 74 (0. 68, 0. 81) | | 0. 65 (0. 57, 0. 71) | 0. 74 (0. 67, 0. 80) | 0. 72 (0. 65, 0. 79) | 0. 75 (0. 69, 0. 80) | 0. 43 |
| 99^th^ | 0. 79 (0. 69, 0. 88) | | 0. 65 (0. 57, 0. 72) | 0. 76 (0. 69, 0. 85) | 0. 81 (0. 71, 0. 91) | 0. 82 (0. 72, 0. 91) | 0. 18 |
| 99. 6^th^ | 0. 80 (0. 65, 0. 95) | | 0. 61 (0. 47, 0. 74) | 0. 74 (0. 59, 0. 89) | 0. 82 (0. 69, 0. 95) | 0. 78 (0. 67, 0. 88) | 0. 17 |
| **10 to 11 years of age** |  | |  |  |  |  |  |
| Mean ^d^ | 0. 29 (0. 27, 0. 31) | | 0. 30 (0. 28, 0. 32) | 0. 31 (0. 29, 0. 33) | 0. 33 (0. 31, 0. 35) | 0. 34 (0. 32, 0. 36) | 0. 003 |
| 1^st^ | -0. 07 (-0. 17, -0. 03) | | -0. 21 (-0. 28, -0. 14) | -0. 06 (-0. 13, 0. 01) | -0. 20 (-0. 28, 0. 12) | -0. 14 (-0. 21, -0. 06) | 0. 41 |
| 2^st^ | -0. 03 (-0. 09, 0. 02) | | -0. 11 (-0. 18, -0. 06) | -0. 04 (-0. 09, 0. 01) | -0. 10 (-0. 18, 0. 05) | -0. 09 (-0. 14, -0. 02) | 0. 25 |
| 5^th^ | 0. 01 (-0. 02, 0. 05) | | -0. 02 (-0. 06, 0. 02) | -0. 02 (-0. 05, 0. 02) | -0. 01 (-0. 05, 0. 03) | -0. 01 (-0. 05, 0. 03) | 0. 51 |
| 10^th^ | 0. 07 (0. 03, 0. 10) | | 0. 05 (0. 02, 0. 09) | 0. 04 (0. 01, 0. 08) | 0. 05 (0. 02, 0. 08) | 0. 05 (0. 02, 0. 09) | 0. 47 |
| 50^th^ | 0. 30 (0. 27, 0. 32) | | 0. 32 (0. 29, 0. 34) | 0. 32 (0. 29, 0. 35) | 0. 36 (0. 33, 0. 38) | 0. 39 (0. 35, 0. 42) | <0. 001 |
| 85^th^ | 0. 49 (0. 45, 0. 52) | | 0. 53 (0. 51, 0. 57) | 0. 54 (0. 52, 0. 57) | 0. 55 (0. 52, 0. 58) | 0. 56 (0. 53, 0. 59) | 0. 004 |
| 90^th^ | 0. 51 (0. 48, 0. 55) | | 0. 54 (0. 51, 0. 57) | 0. 56 (0. 53, 0. 58) | 0. 56 (0. 54, 0. 59) | 0. 57 (0. 54, 0. 60) | 0. 005 |
| 91^st^ | 0. 51 (0. 48, 0. 54) | | 0. 55 (0. 52, 0. 58) | 0. 56 (0. 53, 0. 59) | 0. 57 (0. 54, 0. 60) | 0. 58 (0. 55, 0. 61) | 0. 002 |
| 95^th^ | 0. 50 (0. 47, 0. 54) | | 0. 54 (0. 50, 0. 57) | 0. 55 (0. 51, 0. 59) | 0. 56 (0. 53, 0. 60) | 0. 55 (0. 51, 0. 59) | 0. 07 |
| 98^th^ | 0. 53 (0. 49, 0. 56) | | 0. 52 (0. 47, 0. 56) | 0. 52 (0. 49, 0. 56) | 0. 52 (0. 48, 0. 56) | 0. 53 (0. 49, 0. 58) | 0. 86 |
| 99^th^ | 0. 52 (0. 47, 0. 57) | | 0. 50 (0. 45, 0. 55) | 0. 52 (0. 47, 0. 57) | 0. 50 (0. 46, 0. 55) | 0. 50 (0. 45, 0. 55) | 0. 46 |
| 99. 6^th^ | 0. 55 (0. 48, 0. 62) | | 0. 46 (0. 39, 0. 52) | 0. 53 (0. 48, 0. 59) | 0. 44 (0. 36, 0. 52) | 0. 43 (0. 36, 0. 52) | 0. 06 |

**^a^** zBMI calculated using the UK 1990 Growth Reference.

^b^ Index of Multiple Deprivation (IMD) 2010 decile one (least deprived) versus ten (most deprived) from the lower super output (LSOA) area of the child’s residence.

^c^ Data from the National Child Measurement Programme.

^d^ Estimated using ordinary least squares regression.

^e^ Estimated using quantile regression with decile one as the reference category; 95% confidence intervals calculated using bootstrapping with 100 replications.
